# Effects of Tobacco

**Published:** 1852-10

**Authors:** 


*Effects of Tobacco.*—What are the effects of tobacco upon the teeth and health, is a question frequently asked, and without entering upon a learned discussion of the matter, the inquirer may be directed to the pharmæcopea, wherein its powerful constituents are stated to be *empyreumatic oil* and *alcotina,* both capable of a concentration into the most powerful poisons.

A drop of the oil placed on the tongue will produce convulsions, tetanus in all its forms, coma with a diminution of the powers of voluntary motion, a universal lethargy, often terminating in death, with other effects equally fatal.

A small proportion of a drop of alcotina will kill a rabbit, and a drop a dog; and with these indisputable facts, it seems strange to doubt the injurious consequences of the use of this narcotic acid poison upon the teeth and gums as well as directly upon every part of the frame. Beyond the unpleasant discoloration of the teeth, and an empyreumatical infection of the breath of those accustomed to its use, its deleterious effects may not for a great length of time be detected, but after long habitual use, the whole system becomes injuriously affected, and although habit may apparently reconcile its action when used moderately, nothing can secure the system from its ultimate absorption and irritative properties when employed in excesses or incautiously. Its action on the nervous system is often immediate, and manifested with great power, particularly under excess in smoking, producing a disturbed action of the heart and arteries, and consequently an improper condition in the circulation; but above all, is it objectionable, as it most violently opposes the laws of health laid down by one of the fathers of medicine, "all irritants and stimulants urge and force to a more vehement, and, consequently, a more rapid outlay of the strength or capacity for exertion; and it is an unavoidable law of organization that that outlay is succeeded by depression; and whatever unduly depresses, whether resulting originally from a stimulant, a narcotic, a sedative, or any other powerful principle, has the effect of lessening improperly the action of the heart and arteries, and it is on this account that neither intoxicating drinks, nor tobacco, nor anything else producing an effect which results in depression, can be recommended for the promotion of health and longevity."

Whatever affects the condition of the general health, particularly of an inflammatory character, will affect the teeth and gums, most often, as an immediate consequence, or as a distant change, attributable to a variety of causes. Such active and powerful agents must produce results in the ratio of their potency and the period and excess of use. An unhealthy gum, without great care is adopted, must produce disease of the teeth, and we are of opinion that tobacco creates more injury in this way, than its friction upon the teeth whilst in the mouth, and the constant change of saliva can possibly obviate, and should, therefore, be generally discountenanced.

